# Small Molecule Receptor Binding Inhibitors with In Vivo Efficacy against Botulinum Neurotoxin Serotypes A and E

**DOI:** 10.3390/ijms22168577

**Published:** 2021-08-09

**Authors:** Alon Ben David, Ada Barnea, Eran Diamant, Eyal Dor, Arieh Schwartz, Amram Torgeman, Ran Zichel

**Affiliations:** Department of Biotechnology, Israel Institute for Biological Research, Ness Ziona 74100001, Israel; alonb@iibr.gov.il (A.B.D.); adabarnea@gmail.com (A.B.); erand@iibr.gov.il (E.D.); eyalo@iibr.gov.il (E.D.); ariehs@iibr.gov.il (A.S.); amit@iibr.gov.il (A.T.)

**Keywords:** Botulinum neurotoxin, small molecule inhibitors, high throughput screening, antitoxin

## Abstract

Botulinum neurotoxins (BoNTs) are the most poisonous substances in nature. Currently, the only therapy for botulism is antitoxin. This therapy suffers from several limitations and hence new therapeutic strategies are desired. One of the limitations in discovering BoNT inhibitors is the absence of an in vitro assay that correlates with toxin neutralization in vivo. In this work, a high-throughput screening assay for receptor-binding inhibitors against BoNT/A was developed. The assay is composed of two chimeric proteins: a receptor-simulating protein, consisting of the fourth luminal loop of synaptic vesicle protein 2C fused to glutathione-S-transferase, and a toxin-simulating protein, consisting of the receptor-binding domain of BoNT/A fused to beta-galactosidase. The assay was applied to screen the LOPAC1280 compound library. Seven selected compounds were evaluated in mice exposed to a lethal dose of BoNT/A. The compound aurintricarboxylic acid (ATA) conferred 92% protection, whereas significant delayed time to death (*p* < 0.005) was observed for three additional compounds. Remarkably, ATA was also fully protective in mice challenged with a lethal dose of BoNT/E, which also uses the SV2 receptor. This study demonstrates that receptor-binding inhibitors have the potential to serve as next generation therapeutics for botulism, and therefore the assay developed may facilitate discovery of new anti-BoNT countermeasures.

## 1. Introduction

Botulinum neurotoxins (BoNTs) are the most poisonous substances in nature [1]. These toxins are produced by the Gram-positive spore-forming bacterium *Clostridium botulinum*. There are at least seven serotypes of BoNTs (A–G), of which A, B, E, and rarely F are the cause of botulism in humans [2]. The serotypic nomenclature of BoNTs is based on the observation that antiserum generated against one toxin serotype does not neutralize the toxic effects of another serotype [3]. BoNTs are ~150 kDa proteins produced by the bacterium as a single polypeptide and thereafter proteolytically nicked to form a 100 kDa heavy chain (HC) and a 50 kDa light chain (LC) that are connected together by a disulfide bridge. All BoNT serotypes share a common architecture that consists of three domains responsible for the different steps in the intoxication mechanism: (1) The receptor-binding domain, located on the C-terminus of the heavy chain (also known as the H_C_ fragment); (2) The translocation domain, located on the N-terminus of the heavy chain (H_N_); and (3) The catalytic domain, located on the light chain (LC) [4]. Following exposure, BoNTs bind to specific receptors on motor neurons, and after endocytosis, the LC translocates into the cell cytoplasm, where it cleaves one of three soluble N-ethylmaleimide-sensitive factor attachment protein receptor (SNARE) proteins depending on the BoNT serotype. Cleavage of the SNARE protein prevents neurotransmitter release to muscle cells. This results in flaccid muscle paralysis and can lead to respiratory failure and eventually death [5].

The therapeutic strategies to fight toxins are diverse. Among them are neutralizing antibodies [6,7], antibody fragments [8,9], small scaffold protein binders [10] and small molecule inhibitors [11,12]. These were successfully applied to inhibit shiga toxin, diphtheria toxin, anthrax toxin, ricin, and other toxins. Currently, the only approved therapy for botulinum intoxication is antitoxin, an antibody preparation mostly produced from vaccinated horses [13], which neutralizes the toxin in the bloodstream. For the treatment of infant botulism a human-derived antitoxin preparation is also available in the United States (BabyBIG) [14]. Although effective, equine antitoxin suffers from several drawbacks. First, the administration of a large dose of a foreign protein can cause severe side effects, including anaphylactic shock. Furthermore, antitoxin can be administered for only one intoxication event per patient due to the risk of a secondary immunological reaction against the equine antibodies. For these reasons, antitoxin is given only after the manifestation of the clinical symptoms of botulism. Second, antitoxin therapy is expensive due to the requirements for horses and restricted safety facilities dictated by working with hazardous neurotoxins. Third, antibodies are thermally labile and require cold chain delivery, which limits antitoxin distribution [15,16]. For these reasons, there is motivation to develop next-generation therapies for botulism. One interesting therapeutic approach is small molecules that would inhibit the neurotoxins. Small molecules are appealing for a variety of reasons. The production costs of small molecules are relatively low. The immune system does not react against small molecules and therefore, such therapy can be safe and administered repeatedly and even prophylactically. Moreover, small molecules are generally stable and do not require the complexity involved with cold chain delivery.

The target for small molecule therapeutics can be each of the neurotoxin domains, which are responsible for the different intoxication steps, i.e., receptor binding, toxin translocation, and enzymatic proteolysis of cytoplasmic SNARE proteins. Considerable efforts have been made thus far to find small molecule inhibitors (SMIs) of the catalytic domain in order to inactivate intracellular toxins [16,17,18]. Indeed, several small molecules were found to efficiently inhibit the catalytic domain of BoNTs, with dissociation constants in the range of several dozen nanomolar. Nevertheless, only limited therapeutic effects have been reported thus far in vivo for these catalytic domain inhibitors, most likely due to their lack or limited ability to enter neural cells and reach the site of BoNT action [16,17,19].

In contrast to catalytic domain inhibitors, targeting the H_C_ fragment binding to its receptor could be an attractive option since the molecule needs to encounter the toxin or the receptor outside the target cells. Nevertheless, unlike the major efforts spent in finding inhibitors for the light chain, this approach has barely been explored. One of the reasons for this lack of investigation is the absence of an in vitro assay that readily measures the interaction between BoNT and its receptors.

In humans, BoNT/A intoxication poses a great threat since it is considered the most potent among all BoNTs, with the longest duration of paralysis [20]. To address potential small molecule therapy for BoNT/A, we herein describe the development of a high-throughput screening (HTS) assay that monitors the binding of the receptor binding domain of BoNT/A to its protein receptor, synaptic vesicle protein 2C [21,22]. This unique assay is simple, fast, specific, direct, and uses standard laboratory equipment. An additional important advantage is that this assay circumvents the use of hazardous neurotoxins and is therefore not confined to laboratories with high biosafety levels. This assay was applied to screen a compound library (LOPAC^®^1280) containing 1280 pharmaceutically active compounds and FDA-approved drugs for BoNT/A inhibition. Selected compounds exhibited significant therapeutic effects in mice challenged with a lethal dose of BoNT/A and BoNT/E.

## 2. Results

### 2.1. High-Throughput Screening Assay for Inhibitors of BoNT/A-SV2C Binding

**Figure 1:**
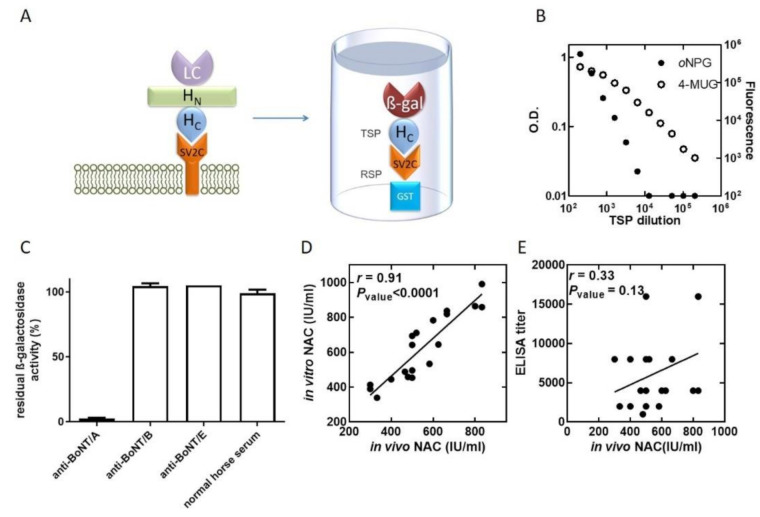
TSP-RSP binding assay. (**A**) Botulinum neurotoxin A consists of three domains: the receptor binding domain (H_C_ fragment), the translocation domain (H_N_ fragment), and the catalytic domain (LC). The toxin binds to the membrane protein receptor SV2C on neural cells. The TSP-RSP binding assay makes use of two chimeric proteins, RSP adsorbed to 96-well plates and the bound TSP detected by measuring beta-galactosidase activity. (**B**) TSP was serially diluted and incubated with an RSP-coated 96-well plate. Bound TSP was detected by development with either the chromogenic substrate *o*NPG or the fluorogenic substrate 4-MUG. By using a fluorogenic substrate, the TSP-RSP binding assay enables the detection of a low TSP concentration equivalent on a molar basis to approximately 6 times the LD_50_/_mL_. (**C**) Prevention of TSP-RSP binding by BoNT/A antitoxin. TSP was incubated with horse anti-BoNT/A, anti-BoNT/B, or anti-BoNT/E antibodies or naïve serum, and then the mixtures were transferred to 96-well plates coated with RSP. Following incubation and washing to remove unbound TSP, the bound TSP was detected by addition of the substrate *o*NPG. Horse anti-BoNT/B, anti-BoNT/E and naïve horse serum did not reduce TSP binding to RSP, while horse anti-BoNT/A reduced the amount of bound TSP by 97.5%. (**D**) The in vitro TSP-RSP binding assay exhibited a high correlation with the in vivo mouse neutralization assay. The neutralizing antibody concentration (NAC) of 20 plasma samples was determined using the pharmacopoeial mouse neutralization assay and the in vitro TSP-RSP binding assay. High correlation was obtained between the two methods. On the other hand, the correlation between the neutralizing antibody concentration determined using the pharmacopoeial mouse neutralization assay and the ELISA titers of the samples was poor (**E**).

The entrance of BoNT/A into neurons begins with the interaction between the HC-fragment domain of the toxin and the synaptic vesicle protein SV2 on neuronal cells, with the highest affinity toward the fourth luminal loop of variant SV2C [21] (Figure 1A). To carry out a direct screening for BoNT/A-SV2C interaction inhibitors, a high-throughput screening (HTS) assay for discovering small molecules was developed. To this end, the essential participating determinants of each interacting protein were assembled into chimeric proteins (Figure 1A, right panel). The H_C_ fragment of BoNT/A was fused to the reporting enzyme beta-galactosidase to produce toxin-simulating protein (TSP). The fourth luminal loop of SV2C was fused to glutathione-S-transferase (GST), resulting in receptor-simulating protein (RSP). Both recombinant chimeric proteins were expressed and produced from *Escherichia coli*. The assay was conducted in 96-well plates, where RSP was adsorbed to the wells and the bound TSP molecules were detected by the enzymatic activity of beta-galactosidase.

Several considerations for the inhibitor screening assay were addressed. First, we wished to design an assay with TSP concentrations comparable to the BoNT/A concentrations expected in patients. Typically, in botulism patients, the detected BoNT concentration in serum samples is in the range of several times the mouse LD_50_ per milliliter [23,24]. The sensitivity of the assay was evaluated by incubating serial dilutions of TSP with RSP-coated plates. Following removal of the unbound TSP, the chromogenic beta-galactosidase substrate *ortho*-nitrophenyl-ß-galactopyranoside (*o*NPG) was added, which allowed the detection of 5 ng/mL TSP, which is equivalent to approximately 200 times the LD_50_/_mL_. In an attempt to improve the assay sensitivity, the fluorescent substrate 4-methylumbelliferyl-ß-galactopyranoside (4-MUG) was applied. The use of 4-MUG dramatically improved the sensitivity, and the detection of 160 pg/mL TSP, equivalent to approximately 6 times the LD_50_/_mL_, was achieved (Figure 1B). Thus, as it enabled higher sensitivity, 4-MUG was used for the compound library screening.

The main mechanism by which immunoglobulin-based antitoxins neutralize BoNTs is by preventing receptor binding, and, thus, most of the neutralizing antibodies are directed toward the H_C_ fragment [25]. Therefore, to validate the compatibility of the assay to detect inhibition of RSP-TSP binding and assess its specificity, the response to equine antitoxins A, B and E was tested. TSP was incubated with botulinum antitoxin A, B or E, and then the mixtures were allowed to interact with RSP (Figure 1C). Only botulinum antitoxin A significantly inhibited the interaction, while inhibition was not observed for botulinum antitoxins B and E.

Antitoxin is currently the only approved therapy for botulism. The potency of antitoxin preparations is determined using the pharmacopoeial mouse neutralization assay (MNA). In this test, the antibodies are diluted and mixed with a constant test dose of the toxin. Following incubation, the mixtures are injected into mice, and survival is monitored. Antibodies binding to the toxin by itself, as expressed by enzyme-linked immunosorbent assay (ELISA) titers, is known to have a poor correlation to the MNA [26] because not all binding antibodies neutralize the toxin. Since the TSP-RSP binding assay measures the capacity of an agent to prevent BoNT binding to the receptor SV2C, we reason that this inhibition could be correlated with toxin neutralization determined by the MNA. To test this hypothesis, the inhibition of TSP-RSP binding by plasma samples of horses vaccinated against BoNT/A was measured. The inhibition was translated into neutralization units (international unit/mL (IU/mL), where 1 IU neutralizes at least 10,000 times the LD_50_) using a standard curve of antitoxin with known potency. Indeed, poor correlation was obtained between the ELISA titers of the same plasma samples and the neutralizing antibody concentration determined by the MNA (Figure 1E), as ELISA measures both neutralizing and non-neutralizing antibodies. In contrast, a high correlation coefficient (*r* = 0.91, *p* < 0.0001) was obtained between the NAC determined by the TSP-RSP in vitro assay and by the MNA in vivo assay (Figure 1D). These results further validate the compatibility of the TSP-RSP assay to find new botulism countermeasures.

### 2.2. Screening of the Compound Library

**Figure 2:**
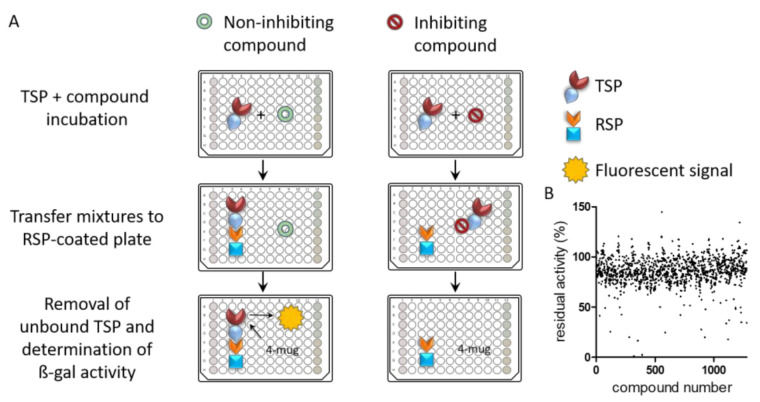
(**A**) Schematic illustration of LOPAC1280 screening using the TSP-RSP assay. Compounds and TSP were mixed, and after incubation, the mixtures were transferred into an RSP-coated plate. Unbound TSP was removed by washing, and residual ß-gal activity on 4-MUG was monitored. Incubation with inhibiting compounds resulted in a reduced fluorescent signal. Each plate included internal controls: no TSP wells (**red**), no compound wells (**green**), and inhibiting antibody wells (**yellow**). (**B**) Activity distribution of each compound based on the residual activity average of the screening replicates for each compound.

Having demonstrated that the TSP-RSP assay is suitable for large-scale screening and is indicative of toxin neutralization, we used it to screen the Library Of Pharmacologically Active Compounds (LOPAC) for BoNT/A-SV2C binding inhibitors. This compound library contains 1280 active compounds, of which some are approved drugs. These compounds have diverse pharmacological activities and include ligands, agonists, antagonists, modulators, antibiotics, etc. Each compound was first incubated with TSP, and the mixtures were then transferred to 96-well plates coated with RSP (Figure 2a). Following removal of unbound TSP, beta-galactosidase activity was determined and compared to control wells with 100% activity to determine the inhibition level of each compound (Figure 2A). A typical HTS assay dynamic range profile of the residual activity values was obtained (Figure 2B), and the average Z’-factor of the plates was 0.75. Compounds that reduced the relative binding below a threshold of 30% residual activity were chosen for further analyses.

Out of the 1280 compounds in the library, 8 inhibited TSP-RSP binding beyond a 70% threshold (Table 1). Two of the compounds were ß-lactam antibiotics (cephalosporin C zinc salt and cefotaxime), which share a similar R1 group. Another two are related to the neurological system. Benserazide is a dihydroxyphenylalanine (DOPA) decarboxylase inhibitor that is used in combination with L-DOPA for the management of Parkinson’s disease (under the brand name Madopar). 6-Hydroxy-DL-DOPA (6-OHD) is a precursor of the catecholaminergic neurotoxin 6-hydroxydopamine [27]. The compound aurintricarboxylic acid (ATA) is a DNA topoisomerase II inhibitor, and protoporphyrin IX is an activator of soluble guanylyl cyclase [28]. Isoxanthopterin is a product of xanthine oxidase, formed during pterin oxidation and normally present in bodily fluids, such as plasma and urine. Pyridostatin is a synthetic molecule that binds and stabilizes G-quadruplexes. Notably, we could not find previous references for pharmacologic activity toward botulinum toxin for any of these compounds.

Dose–response analysis was performed to determine the compounds’ IC_50_ values (Table 1 and supplemental file). ATA and 6-OHD exhibited the greatest inhibitory properties, with nearly complete prevention of TSP-RSP binding and the lowest half maximal inhibitory concentration (IC_50_ values of 1–2 µM). Benserazide inhibited 95% of the TSP-RSP interaction, but its IC_50_ was an order of magnitude higher (18 µM). For both antibiotics, similar minimal relative binding was achieved at their highest concentration (~90% inhibition), with cefotaxime having a lower IC_50_ than cephalosporin C. Both PPIX and pyridostatin inhibited TSP-RSP binding by approximately 80%, and isoxanthopterin exhibited 68% inhibition at 0.1 mM.

### 2.3. Therapeutic Effects of the Selected Inhibitors against BoNT/A Challenge

**Figure 3:**
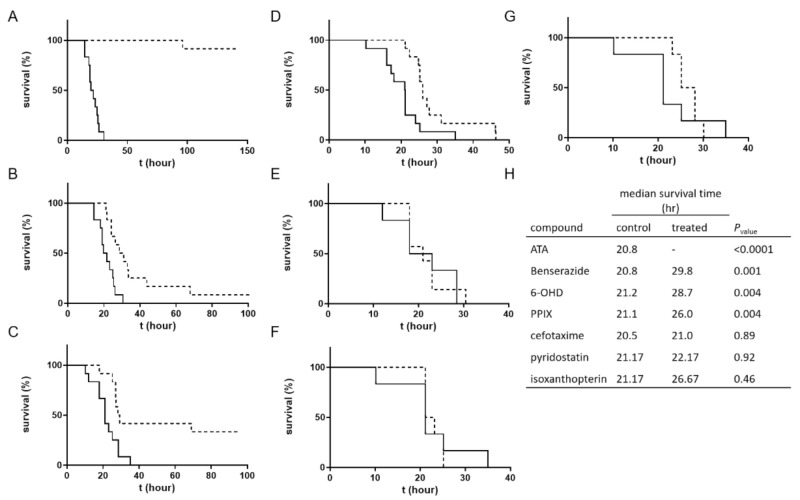
Therapeutic effects of hit compounds in a mouse model. Mice (n = 6) were administered a toxin dose of 4 times the LD_50_ and then administered the test compound followed by survival monitoring. The survival curves of the treated animals (dashed lines) were compared to those of a control group injected with solvent (solid lines). Compound doses were (**A**) ATA, 3.125 mg/mouse; (**B**) benserazide, 12.5 mg/mouse; (**C**) 6-OHD, 2.5 mg/mouse; (**D**) PPIX, 1 mg/mouse; (**E**) cefotaxime, 20 mg/mouse; (**F**) pyridostatin, 1 mg/mouse, and (**G**) isoxanthopterin, 4 mg/mouse. (**H**) Comparison of the survival curves was conducted with the log-rank (Mantel–Cox) test. Significant beneficial therapeutic effects were observed for ATA, benserazide, 6-OHD, and PPIX over solvent in two independent experiments.

A reliable method to estimate the therapeutic effectiveness of a selected inhibitor is by testing its activity against a challenge with a lethal dose of toxin in an animal model. Therefore, a mouse model was established to evaluate the pharmacological activity of the compounds. The toxin dose used for the challenge was chosen to ensure no survival in the untreated control group [29] while still keeping it low enough to avoid masking therapeutic effects. Mice were administered a lethal toxin dose of 4 times the LD_50_ into the left side of the peritoneum, and the compounds were subsequently administered to the right side. The doses of the compounds were determined based on a preliminary toxicity evaluation where the highest dose that was found to be safe in mice when injected alone was selected to be tested in the efficacy assay. Following toxin and compound administration, survival was monitored for four days. The survival curves of the treated animals were compared to that of a control group injected with solvent only (Figure 3).

Four of the examined compounds exhibited significant therapeutic effects. Eleven of the twelve (91.6%) mice treated with ATA were fully protected, and the death of the nonsurviving animal was delayed from a median survival time of 20.75 h in the control group to 96 h (*p*-value < 0.0001). For benserazide, the median survival time was significantly delayed from 20.8 h in the control group to 29.8 h in the treated group (*p*-value = 0.0011). Four animals in the 6-OHD-treated group survived the challenge (33%), and the median survival time for the nonsurviving animals was delayed from 21.2 h in the control group to 28.7 h (*p*-value = 0.004). A significant delay was also obtained in the TTD of the PPIX-treated mice (delayed from 21.1 h in the control group to 26.0 h in the treated group) (*p*-value = 0.004). The compounds cefotaxime, pyridostatin, and isoxanthopterin did not exert beneficial therapeutic effects on the treated animals.

It should be mentioned that when conducting the TSP-RSP assay, the source of the compounds was the LOPAC1280 library. For animal testing, neat compounds were used. As a quality control means, neat compounds were tested by the TSP-RSP assay. The antibiotics cefotaxime and cephalosporin C failed to inhibit the TSP-RSP interaction, using more than one batch. The reason for the difference between the inhibitory properties of the library stock solutions and the neat compounds is unknown. Since cefotaxime did not exhibit any therapeutic effects, the antibiotic cephalosporin C was not tested in animals.

### 2.4. Therapeutic Effects of the Inhibitors against BoNT/E Challenge

**Figure 4:**
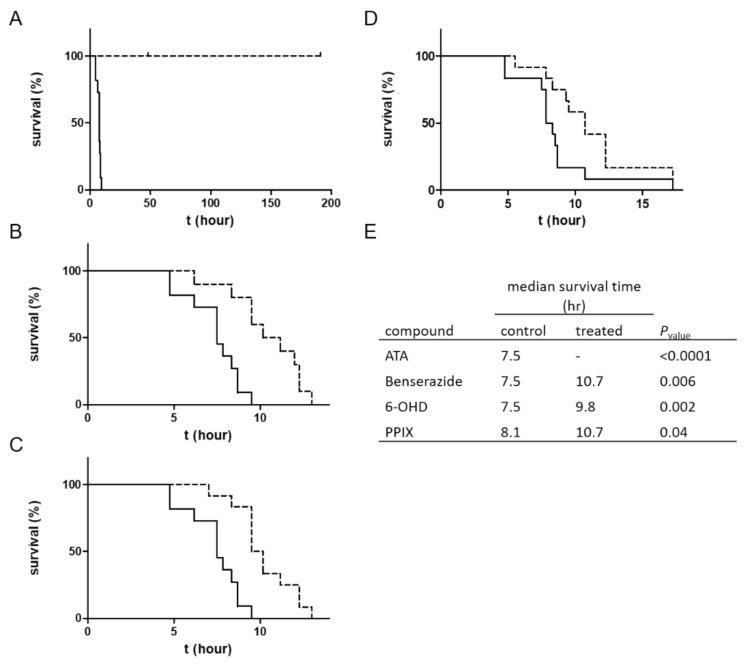
Therapeutic effects of ATA, benserazide, 6-OHD, and PPIX against BoNT/E. Mice (n = 6) were administered a BoNT/E dose of 4 times the LD_50_ and then administered the test compound followed by survival monitoring. The survival curves of the treated animals (**dashed lines**) were compared to those of a control group injected with solvent (**solid lines**). Compound doses were (**A**) ATA, 3.125 mg/mouse; (**B**) benserazide, 12.5 mg/mouse; (**C**) 6-OHD, 2.5 mg/mouse; and (**D**) PPIX, 1 mg/mouse. (**E**) Comparison between the survival curves of mice treated with compounds or with solvent was conducted using the log-rank (Mantel–Cox) test. The results represent two independent experiments.

The serotypic divergence of BoNTs dictates the use of serotype-specific antitoxins toward each BoNT serotype. However, different BoNT serotypes utilize similar protein receptors to identify their target cells. The protein receptors for BoNT/A, BoNT/D, BoNT/E and BoNT/F are synaptic vesicle protein 2 (SV2), and synaptotagmin is the protein receptor for BoNT/B and BoNT/G [30]. The interaction of different BoNTs with the same protein receptor may share common properties. Thus, a small molecule that interferes with the interaction of a specific BoNT serotype and its protein receptor may interfere with the interaction of the protein receptor with another BoNT serotype. To test this hypothesis, the four active compounds that exhibited beneficial pharmacological activity against BoNT/A were evaluated with BoNT/E, which is one of the three most common serotypes in human botulism cases (Figure 4).

Strikingly, the SMI with the highest protective effect against BoNT/A (ATA) completely protected mice from a lethal dose of BoNT/E. A significant delay in the TTD was also observed for the other three compounds. The median TTD of mice treated with benserazide and 6-OHD was delayed from 7.5 h to 10.7 (*p*-value = 0.006) and 9.8 (*p*-value = 0.002) hours, respectively. For mice treated with PPIX, the median survival time was delayed from 8.1 h to 10.7 h (*p*-value = 0.04).

## 3. Discussion

The use of small molecule inhibitors may overcome the current limitations of antitoxin therapy for botulism. Thus far, most efforts have been focused on active-site inhibitors aimed at treating the chronic phase of botulism, where the LC is found within the target cells and the antitoxin is no longer effective. Several potent inhibitors have been reported with *K*_i_ values in the range of several dozen nanomolar [16,17]. The kinetic properties of such inhibitors are usually determined by in vitro enzymatic assays that make use of synthetic substrates such as SNAPtide [31] and BoTEST [32]. Thus far, a reported in vivo therapeutic effect by this promising strategy was mostly a delay in the time to death [17,33]. Further research is needed to allow cell entry of these inhibitors. In this work, we focused on small molecules that inhibit BoNT/A binding to its protein receptor SV2C. The advantage of this approach is that receptor binding inhibitors do not have to enter intoxicated neurons. We believe this approach has hardly been explored due to the lack of a simple assay for measuring the BoNT/A-SV2C interaction. Here, we report the development of the TSP-RSP binding assay as a simple method that aims directly to measure the BoNT/A-SV2C interaction. This assay uses only the protein domains that participate in the natural toxin–receptor interaction. The assay also uses nontoxic components (TSP and RSP) and therefore can be conducted in a biosafety level 1 laboratory. The substrates used to measure beta-galactosidase activity are of relatively low cost, a factor that can be significant when conducting a HTS project of large chemical libraries. Moreover, the signal obtained by beta-galactosidase can be read by basic ELISA readers and special instrumentation is often used to analyze protein–protein interactions, such as surface plasmon resonance and isothermal titration calorimetry, is not required. Most importantly, this assay is indicative of the required property, i.e., BoNT neutralization, and a high and statistically significant correlation was demonstrated between the in vitro and in vivo determination of neutralizing antibody concentrations in horse plasma (D).

Using the TSP-RSP assay, a library of 1280 compounds was screened for BoNT/A-SV2C binding inhibitors. This screening yielded several inhibitors, none of which were previously related to BoNTs. Interestingly, among them are benserazide and 6-OHD, whose pharmacological activities are associated with the nervous system. These compounds share structural similarities with the neurotransmitter dopamine. It was recently reported that SV2C, the protein receptor of BoNT/A, is a mediator of dopamine homeostasis, and impaired SV2C function is linked to Parkinson’s disease (PD) [34]. Due to this high similarity, it is possible that the inhibitory activity of TSP-RSP binding by benserazide and 6-OHA is achieved through interaction with SV2C, thus preventing the H_C_ fragment from binding the receptor. Benserazide is a DOPA decarboxylase inhibitor that is an approved drug. In Europe, benserazide is given together with L-DOPA for the management of PD. In the US, a different DOPA decarboxylase inhibitor named carbidopa is used for that purpose. Notably, in the TSP-RSP binding assay, carbidopa failed to inhibit the interaction between the H_C_/A fragment and SV2C. Another potent inhibitor of the TSP-RSP interaction is ATA. ATA is a polyanionic aromatic compound and a potent DNA topoisomerase II inhibitor [35]. Additionally, it exhibits inhibitory properties against several viruses and bacteria, including *Yersinia pestis* [36], human immunodeficiency virus (HIV) [37], influenza virus [38], and Zika virus [39].

Screening of the LOPAC library revealed several more molecules with different known pharmacological activities. PPIX is a heterocyclic organic compound and a metabolic precursor of hemes, cytochrome C, and chlorophyll [28]. Pyridostatin is a synthetic molecule that selectively interacts with G-quadruplexes, secondary DNA structures found at the telomeric region of the chromosome. Pyridostatin has been investigated as an anticancer drug that specifically promotes growth arrest in cancer cells [40]. Interestingly, PPIX is also known as a G-quadruplex binding molecule and is used as a sensor for G-quadruplex structures [41]. Isoxanthopterin is a product of xanthine oxidase, formed during pterin oxidation and normally present in bodily fluids, such as plasma and urine. The antibiotics cephalosporin C zinc salt and cefotaxime were also found to inhibit TSP-RSP binding. Both antibiotics belong to the beta-lactam group and share the CH_3_COOCH_2_- group at the R1 position. However, due to unknown reasons, the neat states of these antibiotics failed to inhibit TSP-RSP binding.

It is noteworthy that before moving to test the therapeutic effects of the molecules in vivo, we attempted to evaluate these molecules in a cell-based assay. Cellular assays for BoNTs involve all mechanistic steps of intoxication, i.e., binding (to both protein and ganglioside receptors), translocation, and SNAP25 cleavage. Therefore, cellular assays are considered to be more predictive of therapeutic effects than biochemical assays, which usually simulate only a single step of intoxication. The assay used to test the compounds was recently confirmed to determine comparable potency values of antitoxin preparation as the MNA [42]. However, neither of the compounds inhibited BoNT/A in this system. Probable reasons could be related to the fact that unlike the TSP-RSP binding assay, a high toxin dose (1000 times the LD_50_/mL) and prolonged incubations were used in the cell-based assay due to sensitivity limitations. These assay conditions may shift the equilibrium toward receptor binding by the toxin and allows its entrance into the cells, resulting in the cleavage of SNAP25. A similar observation was reported previously by Eubanks et al. for two LC inhibitors that were efficacious in mice and nonetheless showed less effective activity in cellular assays intended to mimic BoNT exposure [43]. Cellular assays with better sensitivity to BoNTs may prove more effective to screen inhibiting compounds. In this regard, it was recently reported by several groups that motor neurons derived from human-induced pluripotent stem cells (hiPSC) exhibit great sensitivity, which even exceeds that of the mouse bioassay [44,45]. Since the secondary cellular assay did not provide us with a filter to further refine our hits and since the number of hits that showed binding inhibition above the set threshold was relatively small and suitable for in vivo testing, the compounds were evaluated next in a mouse model.

In the mouse assay, the compound ATA conferred nearly full protection (92%) in mice intoxicated with 4 times the LD_50_ of BoNT/A. To the best of our knowledge, this is the first report of protection from a lethal dose of BoNT by a small molecule. Benserazide, 6-OHD, and PPIX also exhibited a therapeutic effect after only a single administration of each compound, with increased survival or delayed time to death in treated animals. Remarkably, the in vivo therapeutic effects of the molecules are highly correlated with the in vitro inhibiting properties (IC_50_ and minimal relative binding at 0.1 mM compound, Table 1) of the TSP-RSP interaction. This observation is important from two aspects. First, it demonstrates that receptor binding inhibitors may serve as small molecule therapeutics for botulism. Second, it validates the strength of the simple TSP-RSP binding assay as a predictive tool for in vivo pharmacological activity. It should be noted, however, that the compounds found in this work are of relatively low affinity, which is often a characteristic of hit compounds selected after a single round of screening. We believe that future screening of large compound libraries will pave the way toward the discovery of more potent compounds with high selectivity profile, which together with structure–activity relationship studies may achieve improved therapeutic efficacy.

The TSP-RSP assay make use of the sequence of the fourth luminal domain of *Mus musculus* SV2C, which complies with the in vivo testing of selected compounds in a mouse model. The *Homo sapiens* SV2C sequence differs in this domain from that of *M. musculus* by two amino acids (K558Q and F563L). The assay can be easily modified so the RSP will include the *H. sapiens* sequence. However, in a recent study, Weisemann et al. [46] reported that human and rat SV2C (rat and mouse share similar sequence) exhibit similar binding constants to the receptor binding domain of BoNT/A, and, therefore, compounds selected using the mouse SV2C sequence may inhibit the interaction between BoNT/A and human SV2C as well. 

In terms of multi-serotype protection, the use of small molecules that interfere with receptor binding may simplify the current treatment of botulism therapy. While antibody therapy is serotype-specific and requires different antibody preparations to cover all BoNT serotypes, shared receptors and binding mechanisms by BoNTs may allow broad-serotype therapy. Indeed, administration of ATA to mice intoxicated with a lethal dose of BoNT/E resulted in 100% survival. It was previously reported that BoNT/A and BoNT/E exhibit diverse affinities toward different SV2 isoforms; BoNT/A preferentially binds SV2C, while SV2A and B isoforms are the predominant protein receptors for BoNT/E [21,22,47]. Yet, Pellett et al. reported that hiPCS-derived motor neurons, which expressed predominantly SV2C, are highly sensitive to BoNT/E, suggesting that SV2C may be able to substitute SV2A and SV2B as a receptor for BoNT/E [45]. Our results support a common structural basis for the interaction of BoNT/A and BoNT/E with SV2 isoforms, which allowed broad inhibitory effects by the SMIs. Moreover, the TSP-RSP assay may also be utilized to discover anti BoNT/B countermeasures by simple conversion of the interacting components in the chimeric proteins to H_C_/B fragment and the protein receptor SytII. A cocktail of anti BoNT/A, B, and E SMIs of the toxin–receptor interaction may be used as a simple therapy for the three most common botulinum serotypes in humans.

Taken together, this study demonstrates that receptor binding inhibition by small molecules may be a promising approach for the selection of next-generation anti-botulinum therapeutics, with the potential for broad serotypic protection.

## 4. Materials and Methods

### 4.1. Ethics Statement

All animal experiments were performed in accordance with Israeli law and were approved by the Ethics Committee for Animal Experiments at the Israel Institute for Biological Research (Protocol No. M-11-18).

### 4.2. Materials

All chemicals and the LOPAC1280 compound library were purchased from Sigma-Aldrich (St Louis, MO, USA) unless otherwise stated. Cephalosporin C zinc salt was purchased from Santa Cruz Biotechnology (Paso Robles, CA, USA), 6-hydroxy-DL-DOPA was purchased from Ramidus AB (Sweden), and pyridostatin was purchased from Angene (Nanjing, China). Horse anti-BoNT/A, anti-BoNT/B, and anti-BoNT/E plasma samples were from IIBR. Horse antitoxin A standard of known potency (330 IU/mL) was from IIBR. The standard was calibrated using Botulinum Antitoxin Type A standard (batch 59/021) from the National Institute for Biological Standard and Control [45]. Yeast extract, tryptone and gelatin were obtained from Becton Dickinson and Company (Franklin Lakes, NJ, USA). *E. coli* strains and plasmids were purchased from Novagen (Madison, WI, USA). *C. botulinum* A and E strains were obtained from the IIBR collection. The BoNT/A sequence is similar to that of serotype 62A (accession number M30196). The BoNT/E sequence is similar to that of NCTC11219 (accession number X62683). Toxins were prepared from concentrated culture supernatant grown for 6 days in anaerobic culture tubes.

### 4.3. Protein Expression and Purification

The gene for toxin-simulating protein (TSP) was designed to include beta-galactosidase from *E. coli* BL21 on its N-terminus (gene bank code CAQ30819) and the receptor binding domain of BoNT/A, also designated H_C_ fragment, on its C-terminus (gene bank code M30196, amino acids 872-1296). The two proteins were connected by a flexible linker with the sequence (GGGGS)_3_ and a His6 tag was added to the C-terminus. The receptor-simulating protein (RSP) gene consists of a GST tag on its N-terminus and the fourth luminal domain of *Mus musculus* SV2C (amino acids 545–580, GenBank code AAI37862.1) on its C-terminus. Synthetic genes with optimized codon usage were prepared by GenScript (Piscataway, NJ, USA) and cloned into pET-9a.

To produce proteins, *E. coli* BL21(DE3) harboring pET-9a-TSP or pET-9a-RSP was grown in TB media at 18 °C and 250 rpm for ~40 h, and thereafter, the cells were harvested by centrifugation and disrupted by sonication. TSP and RSP were purified using a HisTrap FF 1 mL column and a GSTrap FF 5 mL column (GE Healthcare), respectively, according to the manufacturer’s instructions.

### 4.4. TSP-RSP Binding Assay

The basic procedure of the TSP-RSP binding assay was as follows. A 96-well plate (Maxisorp, Nunc, Roskilde, Denmark) was coated with 50 µL per well RSP (5 µg/mL) diluted in coating buffer (50 mM Na_2_CO_3_, pH 9.6) and incubated overnight at 4°C. The plate was then washed with wash solution (0.9% NaCl, 0.05% Tween 20) and blocked for one hour at 37 °C with TSTA buffer (50 mM Tris, 0.9% NaCl, 0.05% Tween 20, 2% BSA; 200 µL per well). After washing, the plate was incubated for one hour at 37 °C with TSP diluted with TSTA (50 µL per well). The plate was then washed and incubated with 50 µL of either *ortho*-nitrophenyl-galactopyranoside (*o*NPG) solution (1 mg/mL) or 4-methylumbelliferyl-galactopyranoside (4-MUG) solution (0.5 mg/mL) in Z-buffer (100 mM sodium phosphate, 10 mM KCl, 1 mM MgSO_4_, 50 mM ß-mercaptoethanol, pH 7.0) for one hour at 37 °C, and afterwards, the reaction was stopped with stop solution (1 M sodium carbonate; 50 µL per well). For reactions developed with *o*NPG, the absorbance was measured at 420 nm, and for reactions developed with 4-MUG, the fluorescence was measured (excitation 355 nm, emission 455 nm) using a Synergy HTX plate reader (BioTek Instruments).

For the evaluation of the capacity of horse anti-BoNT/A, anti-BoNT/B, or anti-BoNT/E to prevent TSP-RSP binding, the TSP and the antibodies were incubated for one hour at 25°C (TSP concentration of 10 ng/mL, antibody dilution of 1:3300), and the mixture was transferred to an RSP-coated 96-well plate. Following incubation for one hour at 37 °C and washing, the plate was developed with *o*NPG.

### 4.5. Determination of the Neutralizing Antibody Concentration (NAC)

Plasma samples of horses vaccinated against BoNT/A and antitoxin standard of known potency (330 IU/mL) were serially diluted (dilution factor of 1.5) in TSTA and mixed in a 1:1 ratio with TSP solution (325 ng/mL). The mixture was incubated for one hour at room temperature, transferred to 96-well plates coated with RSP, and then the plate was incubated for one hour at 37 °C. After incubation, the plate was washed with wash solution, and a substrate solution (*o*NPG) was added. Following incubation for one hour at 37 °C, the reaction was stopped by the addition of stop solution, and the absorbance at 420 nm was measured. The NACs in the unknown samples were determined by interpolation from the known antitoxin standard curve using a 4-parameter logistic regression model.

The in vivo potency of equine plasma samples was determined according to the European pharmacopoeia [46]. One international unit (IU) of antitoxin is defined as the amount of antitoxin that neutralizes at least 10^4^ times the mouse LD_50_.

The ELISA titer for binding antibodies was determined by coating a 96-well plate with H_C_/A fragment [47] solution (10 µg/mL in coating buffer, 50 µL per well) and incubation overnight at 4 °C. Following washing, the plate was blocked with TSTA (200 µL per well, 1 h incubation at 37 °C). The plate was then washed and loaded with serial dilutions of plasma samples (50 µL per well). Following incubation (37 °C, 1 h), the plate was washed and incubated with alkaline phosphatase-conjugated goat anti-horse IgG (Jackson ImmunoResearch). Finally, the plates were washed with wash solution, and the colorimetric reaction was developed using the substrate *p*-nitrophenyl phosphate (1 mg/mL in 0.2 M Tris buffer). Following 15 min of incubation (37 °C), the absorbance was measured at 405 nm. The titer was defined as the highest dilution of the sample for which the signal was above 0.4.

### 4.6. Compound Library Screening

The Library of Pharmacologically Active Compounds (LOPAC) contains 1280 active compounds dispensed in 96-well plates at a concentration of 10 mM in dimethyl sulfoxide (DMSO). The compounds were diluted 10-fold in 1:1 DMSO:PBS to obtain a stock plate with each of the compounds at a concentration of 1 mM.

The screening included the mixing of TSP solution (final concentration 3 ng/mL in TSTA) with the compounds (final concentration 0.1 mM) in a polypropylene 96-well plate (Nunc) and incubation of the mixtures for 1 h at 25 °C. The mixtures were then transferred into 96-well plates coated with RSP (50 µL per well) followed by incubation for 1 h at 37 °C. After washing the plate, it was developed with 4-MUG. The plate controls included blank wells without TSP, 100% activity wells without compounds, and positive control for inhibition wells that included a neutralizing monoclonal antibody [48]. For each well, the residual ß-gal activity was calculated using the equation:

Residual activity = 100 × (WF-BF)/PCF, where WF is the fluorescence of the well, BF is the fluorescence of the blank, and PCF is the fluorescence of the 100% activity control wells.

The IC_50_ of the selected compounds was determined by incubation of TSP solution (final concentration 3 ng/mL in TSTA) with serial dilutions of the compounds (from 0.1 to 4.6 × 10^−5^ mM) for one hour at 25 °C. The mixtures were then transferred to 96-well plates coated with RSP (50 µL per well), and the plates were incubated for 1 h at 37 °C. Following washing, the plate was developed with 4-MUG. The IC_50_ values were determined using a 4-parameter logistic regression model.

### 4.7. Testing of Selected Compounds in Mice

CD-1 mice (Charles River, UK) were exposed to 4 times the mouse intraperitoneal (Ms IP) LD_50_ of BoNT/A or BoNT/E in gelatin buffer (0.5 mL) by injection into the left side of the peritoneum. The selected compounds were subsequently administered to the right side of the peritoneum in a volume of 1 mL (n = 6). ATA, benserazide, 6-OHD and cefotaxime were dissolved in PBS. PPIX is soluble in acidic solution; therefore, it was first dissolved in 1 M HCl and sonicated. The solution was then titrated to pH 7.5 with NaOH, and water was added to obtain a 1 mg/mL solution. A control group was administered 1 mL of PBS. Following toxin and compound injection, the survival of the mice was monitored. Comparison of the survival curves was conducted with the log-rank (Mantel–Cox) test using GraphPad Prism. Differences were considered significant at *p* < 0.05.

## 5. Patents

The Israeli Institute for Biological Research filed two patents related to this work (“methods for identifying anti clostridial neurotoxin compounds” No. PCT/IL2021/050039 and “compounds for use in treatment and/or prevention of clostridial neurotoxins intoxication” No. PCT/IL2021/050040).

## Figures and Tables

**Table 1:** TSP-RSP interaction inhibition properties of hit compounds.

Compound	Structure	IC_50_ (µM) *	Maximal Inhibition (%) **
Aurintricarboxylic acid (ATA)	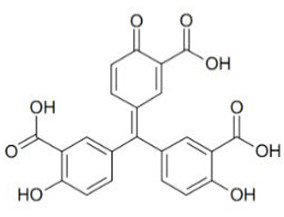	2	100
6-Hydroxy-DL-DOPA (6-OHD)	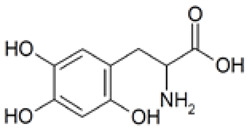	1	98
Benserazide hydrochloride	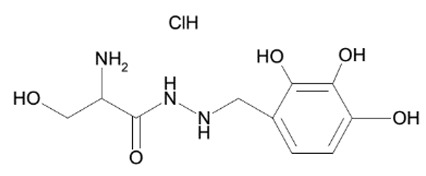	18	95
Cephalosporin C zinc salt	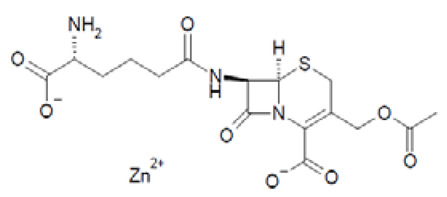	27	90
Cefotaxime sodium	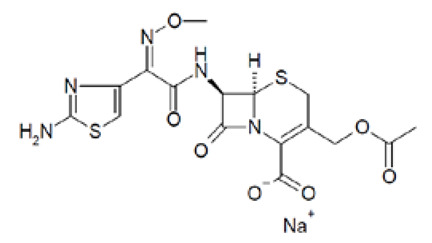	3	91
Protoporphyrin IX disodium (PPIX)	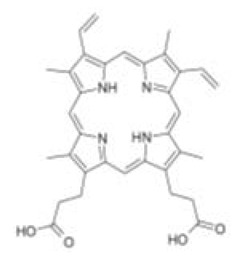	84	81
Pyridostatin trifluoroacetate salt	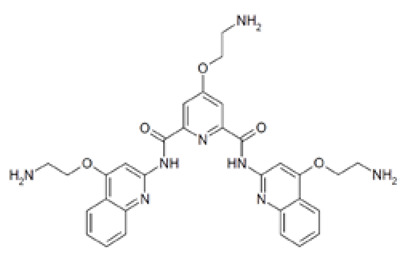	4	77
Isoxanthopterin	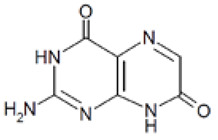	34	68

* IC_50_ curves are presented in a supplemental file. ** The inhibition is compared to control wells containing no compound. Values were calculated by subtracting the relative ß-galactosidase activity at 100 µM compound from the activity in the control wells.

## Data Availability

The data presented in this study are available on request from the corresponding author.

## Supplementary Materials

The following are available online at https://www.mdpi.com/article/10.3390/ijms22168577/s1.
