# Supplementary material for: Small Molecule Receptor Binding Inhibitors with In Vivo Efficacy against Botulinum Neurotoxin Serotypes A and E
Source: Int J Mol Sci. 2021 Aug 9;22(16):8577. doi: 10.3390/ijms22168577 (PMC8395308; doi:10.3390/ijms22168577)

IC50 curves of the selected compounds described in the study.

ATA

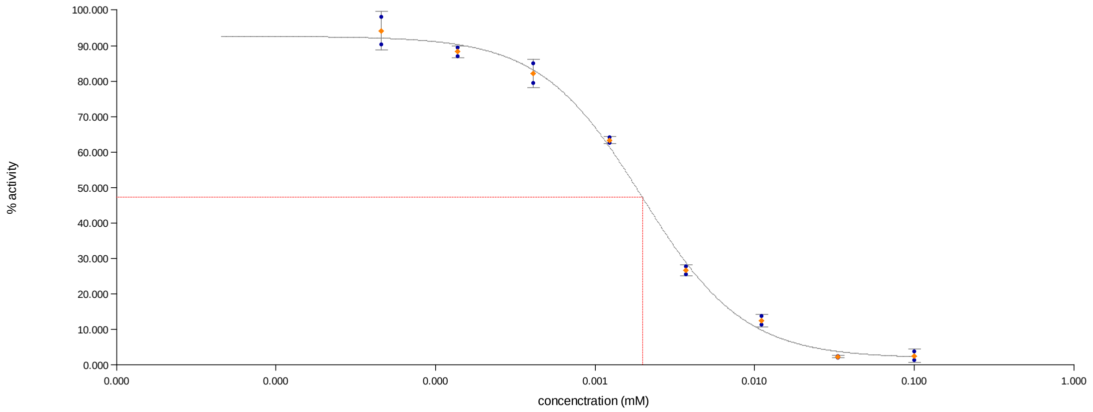

6-OHD

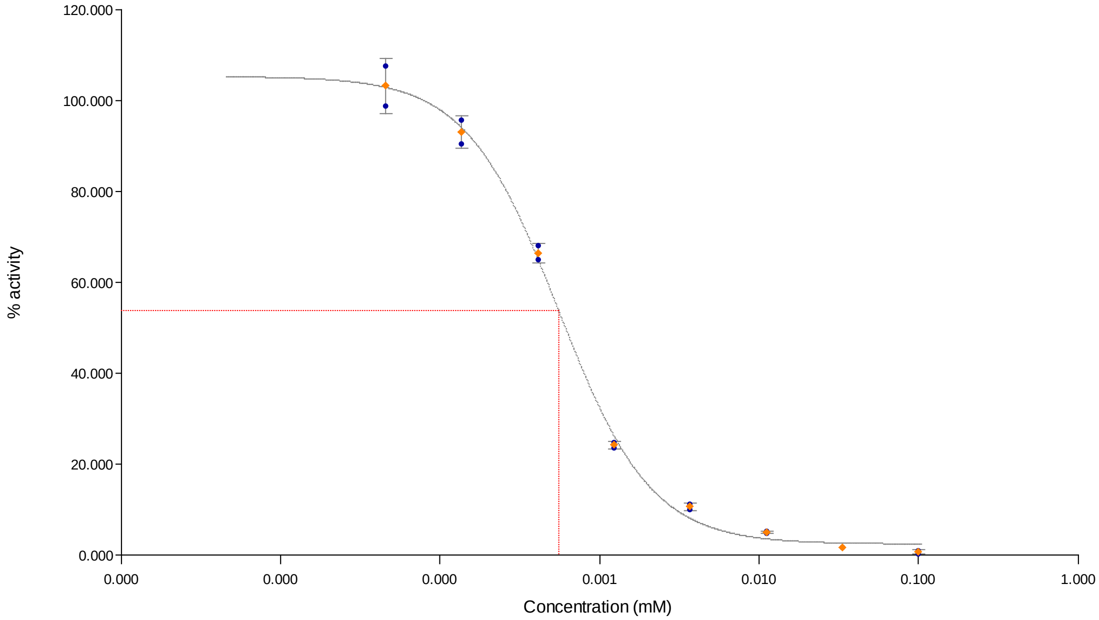

Benserazide

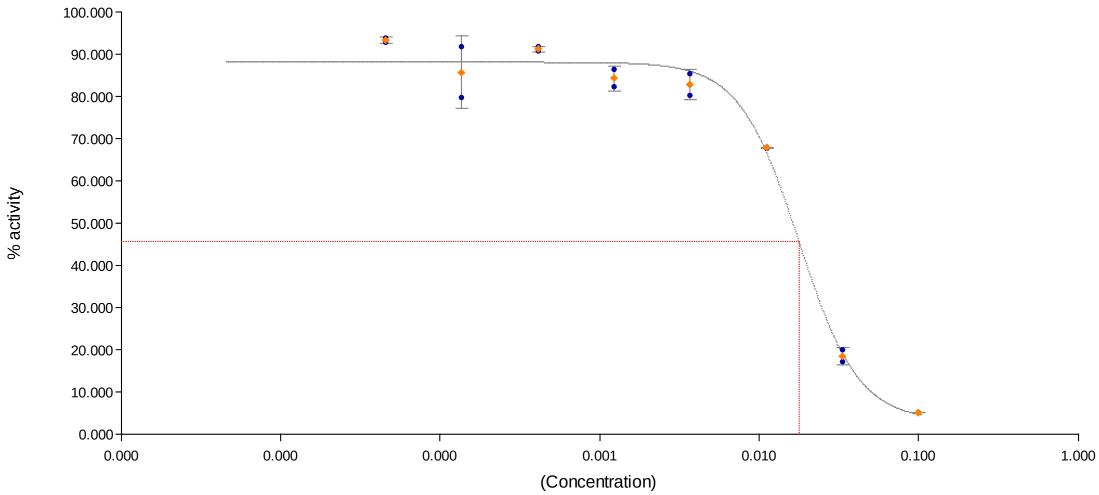

Cephalosporin C zinc salt

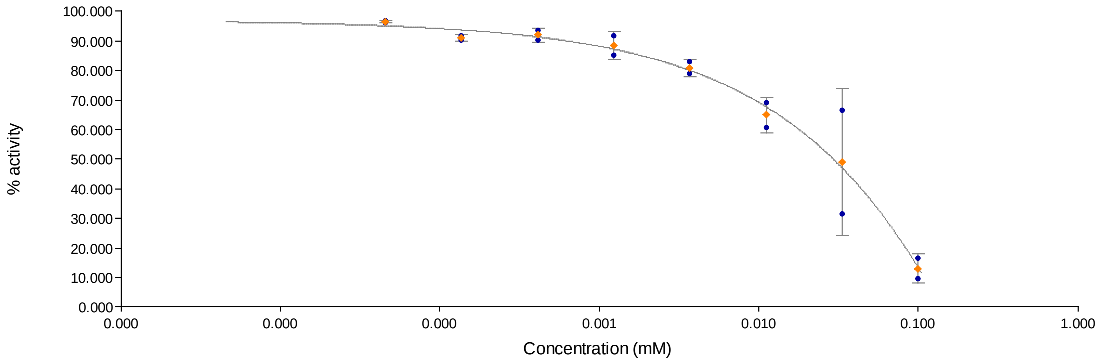

Cefotaxime sodium

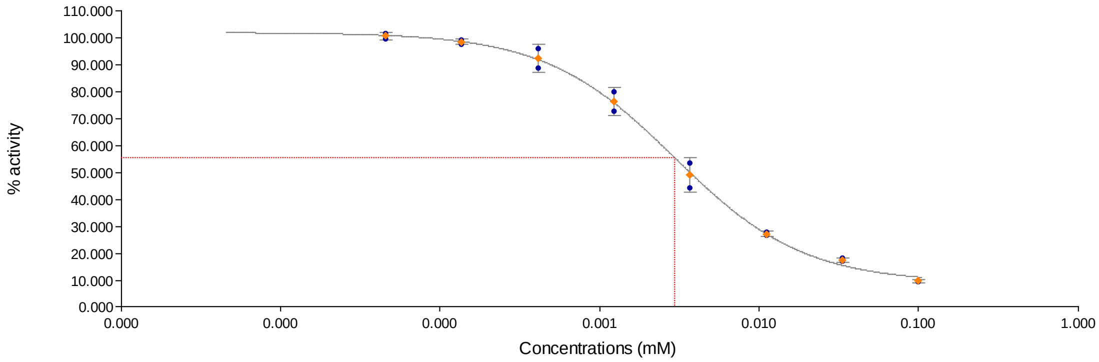

Protoporphyrin IX disodium (PPIX)

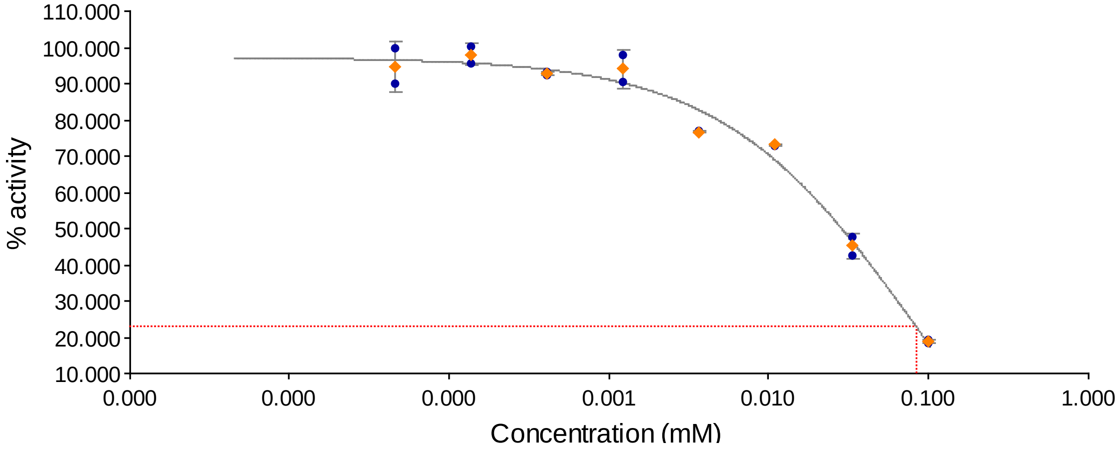

Pyridostatin

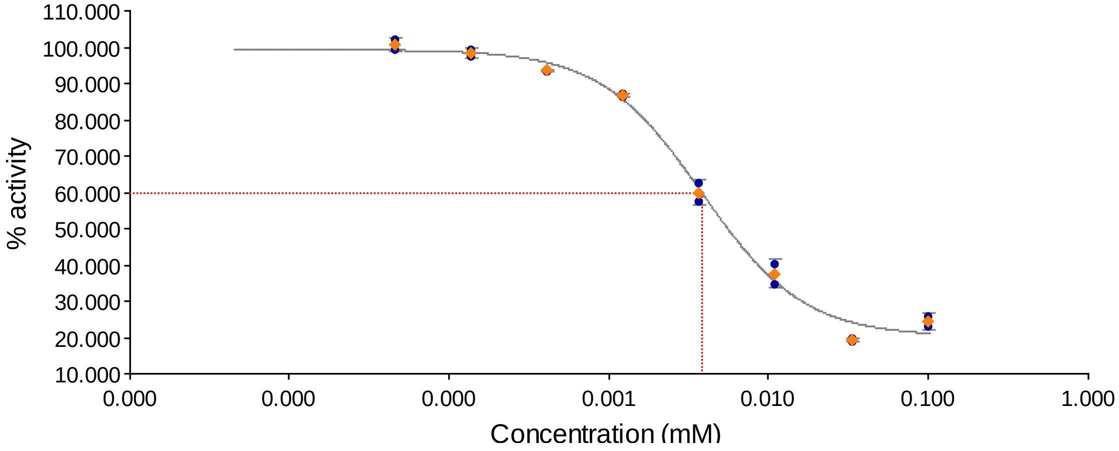

Isoxanthopterin

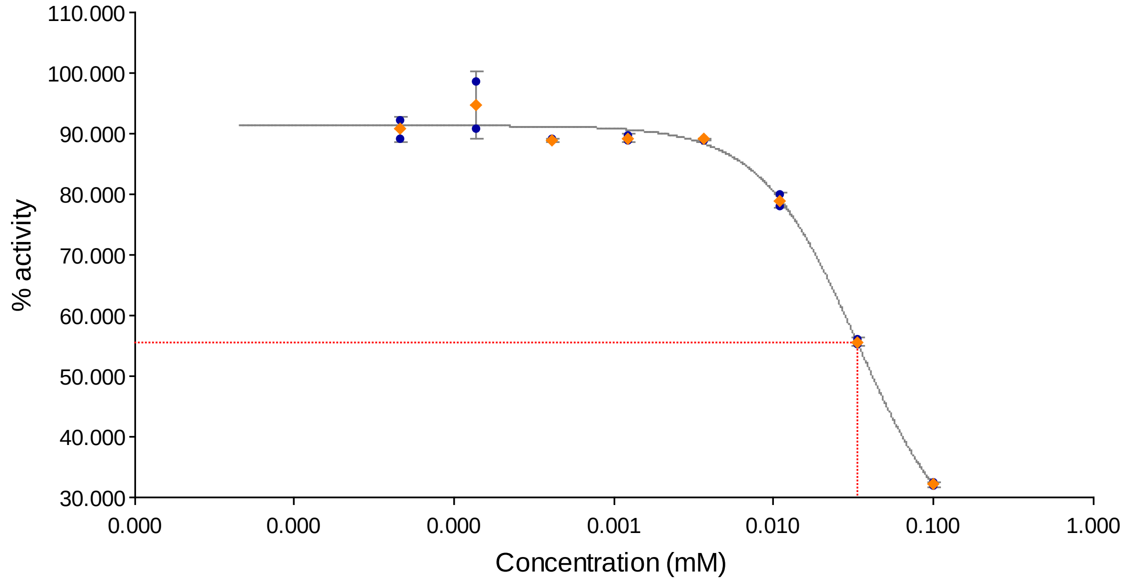

Supplement: Supplementary file 1 [file ijms-22-08577-s001.zip › ijms-1278902-supplementary.pdf]
